# Development of Piezoresistive Sensors Based on Graphene Nanoplatelets Screen-Printed on Woven and Knitted Fabrics: Optimisation of Active Layer Formulation and Transversal/Longitudinal Textile Direction

**DOI:** 10.3390/ma15155185

**Published:** 2022-07-26

**Authors:** Luisa M. Arruda, Inês P. Moreira, Usha Kiran Sanivada, Helder Carvalho, Raul Fangueiro

**Affiliations:** 1 Centre for Textile Science and Technology (2C2T), University of Minho, 4800-058 Guimaraes, Portugal; ines.moreira@fibrenamics.com (I.P.M.); ushakiran.sanivada@gmail.com (U.K.S.); helder@det.uminho.pt (H.C.); rfangueiro@det.uminho.pt (R.F.); 2 Fibrenamics, Institute of Innovation on Fibre-Based Materials and Composites, University of Minho, 4800-058 Guimaraes, Portugal

**Keywords:** smart textiles, piezoresistive sensors, graphene nanoplatelets, carbon-based materials, screen-printing

## Abstract

Although the force/pressure applied onto a textile substrate through a uniaxial compression is constant and independent of the yarn direction, it should be noted that such mechanical action causes a geometric change in the substrate, which can be identified by the reduction in its lateral thickness. Therefore, the objective of this study was to investigate the influence of the fabric orientation on both knitted and woven pressure sensors, in order to generate knowledge for a better design process during textile piezoresistive sensor development. For this purpose, these distinct textile structures were doped with different concentrations of graphene nanoplatelets (GNPs), using the screen-printing technique. The chemical and physical properties of these screen-printed fabrics were analysed using Field Emission Scanning Electron Microscopy, Ground State Diffuse Reflectance and Raman Spectroscopy. Samples were subjected to tests determining linear electrical surface resistance and piezoresistive behaviour. In the results, a higher presence of conductive material was found in woven structures. For the doped samples, the electrical resistance varied between 10^5^ Ω and 10^1^ Ω, for the GNPs’ percentage increase. The lowest resistance value was observed for the woven fabric with 15% GNPs (3.67 ± 8.17 × 10^1^ Ω). The samples showed different electrical behaviour according to the fabric orientation. Overall, greater sensitivity in the longitudinal direction and a lower coefficient of variation CV% of the measurement was identified in the transversal direction, coursewise for knitted and weftwise for woven fabrics. The woven fabric doped with 5% GNPs assembled in the weftwise direction was shown to be the most indicated for a piezoresistive sensor, due to its most uniform response and most accurate measure of mechanical stress.

## 1. Introduction

In addition to the role as a social communication tool [1] and the function of protection against environmental factors [2], textiles, by wrapping the body, can be the “territory” to detect internal (corporeal) and external (environmental) stimuli. This is the concept of Smart Textiles, in other words, fabrics that can interact with the environment/user by detecting and, sometimes, reacting and adapting to mechanical, thermal, chemical or electrical stimuli [3]. One of these stimuli is the pressure that can be exerted by the body itself, through the bones and blood pressure, or through external objects, such as the use of wearable medical devices. In turn, the measurement and action upon said body pressure can, in some cases, prevent the development of skin diseases, like pressure ulcers [4], and cardiovascular diseases, through pulse pressure monitoring [5], as well as being inserted into haptic feedback systems to generate tactile information for amputees [6].

Given their wide applicability, pressure sensors in a wearable textile form can, non-invasively, measure mechanical action on the body that can be used, e.g., to quantify performance or identify possible harm. To meet this sensing requirement, flexible sensors seem promising, as they can mechanically deform, granting them the ability to sense the stimuli at its origin, where the signal is most accurate [7]. Regarding the perspective of comfort, they can also meet the requirements of negligible weight, tailorability to regular daily garments and conformability.

The transduction mechanisms that present electrical responses from mechanical deformations are piezoresistive, piezoelectric, capacitive, triboelectric and transistive [8]. These transduction methods, in general, rely on an active material whose electrical property changes upon mechanical stress. The presence of two electrodes captures this property on the said active material. Among the mechanoelectrical ones, piezoresistive sensors are the most prevalent in the literature to be applied as electronic skin for their simple mechanism, compact structure, low cost, energy consumption and ease of signal acquisition [9].

Sensitivity and detection range are two important parameters to evaluate pressure sensors’ performance. In this context, sensitivity is defined as the rate of change of the electrical property with the change of the measured variable. In bulk piezoresistive sensors, the sensitivity is, generally, measured by the polymer matrix compression modulus and the filler direction change due to decreased percolation pathways caused by disconnections and the change in the compatibility between the conductive materials and the matrix. Furthermore, it should be noted that under uniaxial compression conditions, the load applied to the material will be uniformly distributed over it. However, when referring to an intrinsically anisotropic material, such as a textile substrate, the contact between the fibres in different directions can lead to peculiar deformation performance and, consequently, alter the path of current conduction. In line with this, a recent study carried out by Xie et al. indicated that pressure sensors made of knitted fabric have different sensitivities in the walewise and coursewise directions [10]. This study emphasized that the way the yarns are in contact with each other will influence their compressibility property and, therefore, the electrical response of the sensor. The mechanical performance of a textile substrate will also be influenced by the yarn properties (such as the diameter, the coefficient of friction and the initial Young’s modulus) [11] and by its weave or knit structure.

To our knowledge, no study has encompassed, until now, an analysis of the piezoresistive behaviour in different directions of particular textile substrates. Therefore, this study aims to investigate the influence of the fabric orientation on the performance of knitted and woven pressure sensors, to generate knowledge that enables one to create a better design procedure for the development of textile piezoresistive sensors.

It should be noted that conventional textiles must be functionalised to respond to an electrical stimulus. Most of the time, they become polymeric composites. Thus, to develop the experimental plan of this study, some concepts need to be addressed, namely the working principle of piezoresistive sensors in polymeric composites and the materials employed.

### 1.1. Piezoresistive Behaviour in Polymer Composites

The term piezo comes from the Greek word “piezen”, which means to press and to compress. Through this etymological meaning, it is understood that a piezoresistive sensor transduces an applied pressure into an electrical resistance variation [12].

A simple piezoresistive sensor model consists of an active part positioned between two electrodes. The resistance value of the sensor is defined by being the resistance of the electrodes (R_e_) added to the resistance of the active material (R_a_). It is noteworthy that the change in R_a_ is the reason for the piezoresistive behaviour, considering that R_e_ is a constant value. Through this discursive reasoning, it is worth mentioning the material resistance equation:

(1) $$R=\rho \frac{L}{A}\;$$

where ρ is the material resistivity, L is the length and A is the cross-sectional area.

According to the equation above, and in line with previous studies, the resistance variation in piezoresistive sensors is influenced by two main factors: geometric deformation of the elastomeric composite, where the parameters L and A change according to the material deformation, and the resistivity of fillers in band structure and interparticle separation [9]. The conductive polymer composites, generally composed of conductive particles, i.e., fillers, dispersed in an insulating matrix, rely on the percolation theory. From phase a, known for presenting very low electrical conductivity values, up to zone c, where the filler particles come into direct contact to form perfect networks, the conductivity values increase significantly. At some point, though, the percolation threshold is exceeded, and conductivity does not increase substantially. Hereupon, particle geometries and properties, insulating matrix properties and polymer–particle interaction will directly influence the tunnelling conduction mechanism under a mechanical deformation [9,13].

### 1.2. Materials (Active, Electrodes and Substrates)

Given the above, the materials that compose a sensor have a direct relationship with its piezoresistive response. In its simplest form, the sensor is composed of the sensing material, also known as active material, signal transfer components and a flexible substrate. Each component comprises different requirements.

Active materials are those responsible for detecting the mechanical stimulus. In this sense, they need to possess reliable electrical conduction paths, exceptional chemical stability, good mechanical compliance and compatibility with large-area processing techniques [9]. Semiconducting polymers such as polydimethylsiloxane (PDMS) [14], polypropylene (PP) [15], semiconducting nanowires and carbon-based materials are some of these. However, carbon and its allotropes, such as graphene and nanotubes, are the most used for application in polymer-based composites. In addition, graphene, the single sp^2^-hybridized carbon atom-thick 2D material, has shown to be highly promising due to its excellent mechanical, thermal and electrical properties provided by its internal honeycomb-like structure [16]. Among its variations, graphene nanoplatelets (GNPs) have gained evidence due to their ease of scalability production and low price when compared to others [17]. Furthermore, GNPs have few graphite layers, are lightweight, and have a higher aspect ratio with a planar shape [18]. However, the key challenge in the synthesis and processing of bulk-quantity graphene sheets is aggregation, which tends to occur through van der Waals interactions. In this sense, many synthesis and modification methods have been used, which involve either preparing “amphiphilic” graphene nanoplatelets (a strategy that involves exfoliation followed by Graphene oxide (GO) in situ reductions [19]) or a dry-low-cost method for obtaining N-rich graphene, through gamma irradiation with the use of ethylamine [20]. Furthermore, when dispersed in biopolymers and doped onto textile substrates, such as linen, GNPs’ ecocomposites may have their properties potentiated, namely, improved hydrophobic capacity and UV protection, in addition to their piezoresistive response [21].

Therefore, graphene nanoplatelets were chosen in this study due to their aforementioned superior mechanical, thermal, electrical and scalability properties, beyond the already proven applicability as a filler in polymer matrices with a piezoresistive response.

Regarding signal transfer materials, better known as electrodes, it is expected that they are stretchable materials that can maintain high conductivity under large strains and possess excellent stability [9]. For this purpose, metal electrode materials with a specific design are suitable for stretchable electronics, such as Au, Ag or Cu [22]. Furthermore, in this sense, silver ink was chosen since it has an appropriate electrical conductivity value for electrodes.

For flexible sensors, the substrate can be understood as the base, known as the insulating matrix, in which the active material will be dispersed. To this end, the substrate should have excellent chemical stability, low surface roughness and flexible mechanical properties [7]. Elastomers, self-healing materials, polyurethane (PU) and textile fabrics are presented as some of the materials that meet these requirements.

This work aims to take advantage of the potential of graphene nanoplatelets added to a textile substrate, with its intrinsic characteristics, to produce smart textiles with pressure-sensing properties. Furthermore, we believe that understanding the characteristics and properties of the substrates themselves could be an important tool to orchestrate, in the same sensor, different electrical responses, depending on the specifications required for each application. When it is possible to predict its bi-directional behaviour, such knowledge can strengthen the sensor’s response reliability and, consequently, generate textile sensors that can be applied in society.

## 2. Materials and Methods

### 2.1. Materials

Two distinct textile structures were used, both composed of fibres made of 100% cotton (CO), namely a taffeta woven fabric (Lameirinho, Guimarães, Portugal) and a jersey knitted fabric provided by Impetus (Barqueiros, Barcelos Portugal). Table 1 shows some of their structural characteristics.

GNPs used were provided by Graphenest (Aveiro, Portugal), with 8–30 layers, a thickness of 3–10 nm and layers’ lateral dimensions of 0.5–0.2 µm. To produce an ink paste containing the aforementioned GNPs, the following materials were used: a special polyurethane (PU) finishing agent for a soft hand feel and improved moisture management properties, called Tanapur^TM^ Velluto; Tanapur^TM^ ONE as crosslinker; Edolan XME as dulling concentrate and anti-block agent for aqueous coating applications; as well as the dispersing agent and flow modifier for textile printing and Thickener A02, all from the company Tanatex Chemicals B.V© (ADI Group, Santo Tirso, Portugal). For the electrodes, a commercial conductive ink named Silver100 NP was used, whose conductive component is silver, with an electrical conductivity of <29 mΩ/sq/mil and supplied by the company NanoPaint© (Braga, Portugal). To join the doped textile layers, a thermoplastic bonding net composed of aliphatic polyurethane ester, with 35–50 g/m^2^ and a melting temperature between 109–119 °C, was used (Protechnic, Cernay, France).

### 2.2. Samples Preparation

#### 2.2.1. Sensor Design

**Figure 1:**
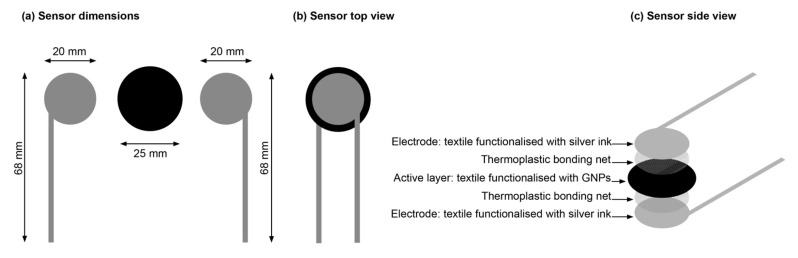
(**a**) Sensor dimensions; (**b**) Sensor top view; (**c**) Sensor side view.

Regarding the pressure/force sensor, its design was carried out in Adobe Illustrator software, having as reference the flexible force sensing resistor (FSR) produced by the company Tekscan (Boston, MA, USA), model FlexiForceTM A201, whose operating principle is the direct force detection. The FlexiForceTM A201 sensor has a 9.53 mm diameter sensing area and a 0.203 mm thickness. However, to improve this detection area, the diameter of the active layer was increased to 25 mm. Figure 1 shows the dimensions of the sensor developed in this study. These drawings were engraved on a screen with 120 threads per inch (TPI), as indicated by the conductive ink manufacturer.

#### 2.2.2. Active Layer Formulation

The second step of this experimental study consisted in the development of a piezoresistive ink paste. For this purpose, different concentrations of the active material were tested, in order to identify the best ratio (% *w*/*v*) for the piezoresistive response. Therefore, 50% Tanapur^TM^ Velluto, 25% Tanapur^TM^ One and 25% Edolan XME were mixed by mechanical stirring at 350 rpm for 30 min. Subsequently, 2%, 3%, 5%, 7%, 10% and 15% (*w*/*v*) of GNPs were slowly dispersed in the previous solution and kept under mechanical stirring for 2 h. After proper GNPs dispersion, 20% (*w*/*v*) of Thickener A02 was added, and the mixture was kept under mechanical agitation at 200 rpm until it acquired the appropriate viscosity of an ink paste.

#### 2.2.3. Screen-Printing and Process

The textile substrates (woven and knitted fabrics) were spread on a Johannes Zimmer screen-printing table. The electrodes were printed with silver ink at a speed of 30 mm/s, followed by a curing process at 120 °C for 10 min on a screen-printing drying oven. Subsequently, the active layer was applied using the previously described GNP paste, at the front and back of the textile substrate; dried for 3 min at 120 °C and cured for 10 min at 160 °C.

**Figure 2:**
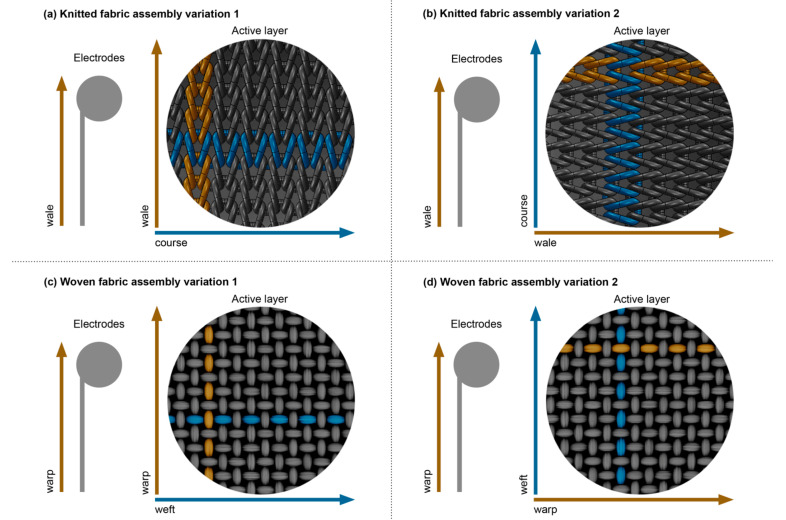
Variations of active layer assembly: (**a**,**c**) the electrodes and the active layer are positioned in the same direction, both in the longitudinal position of the substrates; (**b**,**d**) the electrodes and the active layer are positioned in different directions, with the electrodes in the longitudinal position of the textile substrates and the active layer parallel to the substrates’ transversal position. The copper colour represents the walewise direction in knitted fabrics and the warpwise direction in woven fabrics. The blue colour represents the coursewise direction in knitted fabric and the weftwise direction in woven fabrics.

After the functionalisation of the textiles through the screen-printing technique, the step of joining the layers was carried out. It should be noted that this stage contains variations regarding the direction of the textile substrates. The electrodes were always printed in walewise and warpwise directions, for the knitted and woven fabrics, respectively. The variations refer to the active layer, that is, the positioning direction of the textiles doped with the GNP paste. In this sense, two different position variations were used for each substrate. In variation 1, the walewise (knitted fabric) and warpwise (woven fabric) directions of the active layer were positioned parallel to the walewise and warpwise directions of the textile electrodes, as shown in Figure 2a,c. On the other hand, in variation 2, the coursewise (knitted fabric) and weftwise (woven fabric) directions of the active layer were positioned parallel to the walewise and warpwise direction of the textile electrodes, according to Figure 2b,d. Finally, the layers were cut for each variation and joined through the thermosetting process using the bonding net, at 110 °C, for 10 s, at a pressure of 2.5 bar.

### 2.3. Samples Characterisation

#### 2.3.1. Field Emission Scanning Electron Microscopy (FESEM)

In order to study the impregnation and the GNP’s degree of dispersion in the textile substrates, an FESEM analysis was carried out. The surface morphology of the samples was analysed by FESEM using the NOVA 200 Nano SEM equipment from FEI Company (Hillsboro, OR, USA). All samples were sputter-coated with a palladium-gold (Pd-Au) film (20 nm) to make them conductive. The images were taken in topographic mode with an accelerated voltage of 10 kV, on different scales.

#### 2.3.2. Ground State Diffuse Reflectance (GSDR)

In addition to validating the presence of GNPs in the textile’s fabric, GSDR was performed to identify differences in the absorption spectrum of knitted and woven fabrics, at distinctive concentrations of GNPs. The woven and knitted fabric samples’ GSDR spectra, with and without GNPs, were recorded in the 200 to 800 nm wavelength range, using a Shimadzu UV 2501PC Spectrophotometer. Each sample was analysed in three different places to ensure a reliable analysis. The remission function (F(R)) was calculated according to the Kubelka–Munk equation:

(2) $$F\left(R\right)=\frac{{\left(1-R\right)}^{2}}{2R}=\frac{K}{S}\;$$

where K represents the absorption coefficient, S the dispersion coefficient and R the reflectance.

#### 2.3.3. Raman Spectroscopy

Raman spectroscopy was also carried out to identify the GNP’s presence in the knitted and woven fabrics and their spectra at distinctive concentrations. The woven and knitted fabric samples’ Raman spectra, with and without GNPs, were recorded in the 1400 to 2800 nm wavenumber range, using a Horiba LabRAM HR Evolution confocal microscope (Horiba Scientific, Longjumeau, France), equipped with a 532 nm (2.33 eV) laser.

#### 2.3.4. Linear Electrical Resistance and Electrical Conductivity

**Figure 3:**
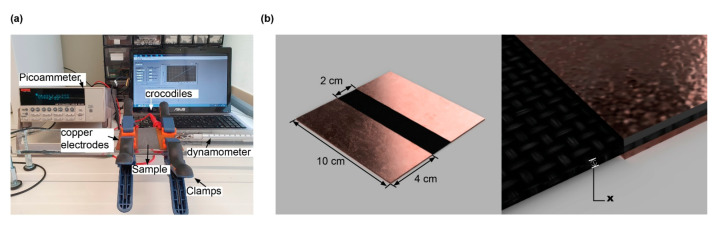
(**a**) Electrical Resistance test set-up (I–V curves); (**b**) sample placement demonstration between four copper electrodes, where x represents the sample thickness.

To measure the linear electrical resistance values of the woven and knitted fabrics, with and without GNPs, the I–V curves (electric current intensity—voltage curves) method was performed. For this purpose, an electrical source (Keithley 487 Picoammeter/Voltage Source), applying a potential difference between −0.8 V to 0.8 V, with a step of 0.1 V at room temperature, was connected to the specimens by conductive electrodes. This set-up was composed of four conductive copper plates with an area of 5 × 10 cm^2^ and an electrode distance of 2 cm, according to Figure 3. The samples have an area of 10 × 10 cm^2^, and they were sandwiched by the electrodes. The electrical resistance values were determined by the slope of the I–V curves.

After the linear electrical resistance values had been obtained, the second Ohm’s law Equation (1), already described in this study, was applied. Electrical resistivity represents the ability of a given material to oppose electric current. Electrical conductivity, expressed in S/m, is the inverse of electrical resistivity, as represented in the subsequent equation:

(3) $$\sigma =\frac{1}{\rho }\;$$

Note that l is 0.02 m, and A  is the thickness multiplied by the sample width. As the characterised samples are 10 × 10 cm^2^, the fixed width is 10 cm, but the thickness varies according to each sample.

#### 2.3.5. Piezoresistive Behaviour

**Figure 4:**
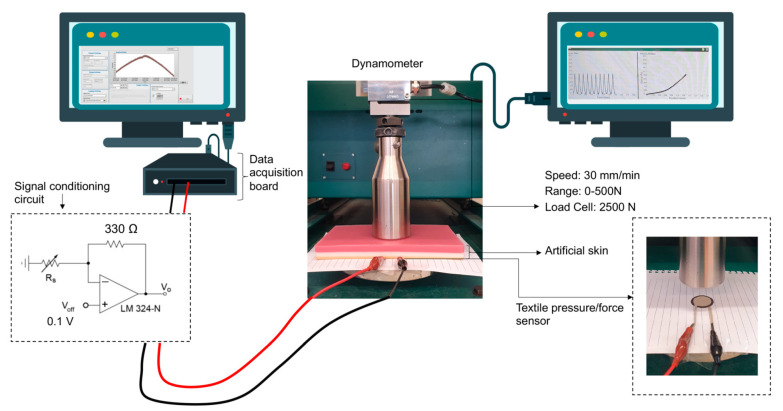
Piezoresistive testing procedure, in which two devices are used simultaneously: a dynamometer, and a signal acquisition circuit.

After manufacturing the pressure/force sensor, the sensors in knitted and woven fabrics were characterised to evaluate their electrical response to mechanical action. To conduct this test, two pieces of equipment were used simultaneously. The first was a universal testing machine, a Hounsfield dynamometer with a 2500 N load cell, performing 10 compression cycles at a speed of 30 mm/min on the samples. As the study encompassed the development of textile-based sensors to be used on the human body, an artificial skin was used to mimic the pressure exerted by the body. This artificial skin was placed between the sample and the load cell. The second equipment was a data acquisition system, composed of a signal conditioning circuit and a National Instruments NI-USB-6229 data acquisition board plugged to a PC running data acquisition software developed in LabVIEW. The signal conditioning circuit was implemented using a non-inverting amplifier configuration with a gain dependent on the sensor resistance R_S_, to which the samples were connected using crocodiles. The complete setup can be observed in Figure 4.

The relationship between sensor resistance, $R_{s}$, and the voltage output, $V_{o}$, is expressed by the following equation:

(4) $$V_{o}=V_{\mathrm{off}}\left(1+\frac{R}{R_{s}}\right)\;$$

where $V_{o}$ represents the output voltage (V), $V_{\mathrm{off}}$ the offset voltage (V), Rs the sensor resistance (Ω) and R the feedback resistance (Ω), which, in our circuit, was 330 Ω. The setup performed in these tests was based on a previous study [23] and provided some linearization of the resistance values produced by typical piezoresistive sensors under compression.

The measurement process provided two separate data files for the 10 compression cycles performed: the force/displacement values, coming from the Universal testing Machine’s software, and voltage/time signals, coming from the signal conditioning/data acquisition system. Another application developed in LabVIEW synchronized the two signals and processed them, providing voltage versus force data. These data were divided into 20 half-cycles and characterised in different ways. In particular, the coefficients of variation (CV%) for the output voltage at each force value were calculated.

The data obtained after synchronisation represented the function

(5) $$V_{o}=f\left(F\right)$$

where $V_{o}$ is the output voltage, and F is the force applied by the testing machine.

At each sample p of this function, the standard deviation $\;\sigma \left(p\right)$ of $V_{o}$ was computed as

(6) $$\sigma \left(p\right)=\sqrt[{2}]{\overset{N-1}{\underset{i=0}{\sum }}\frac{(V_{o}\left(i\right)-{\left(\bar{V_{o}}\left(p\right)\right)}^{2}}{N-1}}$$

with N= number of half cycles of the compression test and $\bar{V_{o}}\left(p\right)=$ average of output voltage in the N half cycles at sample p.

Finally, CV% at sample p was found as

(7) $$\mathrm{CV}\%\left(p\right)=\frac{\sigma \left(p\right)}{\bar{V_{o}}\left(p\right)}\cdot 100$$

To obtain average sensor resistance at sample p, Equation (8) was solved for R_s_:

(8) $$R_{s}\left(p\right)=\frac{R}{\frac{\bar{V_{o}}\left(p\right)}{V_{\mathrm{off}}}-1}$$

In turn, on the basis of these resistance values, sensor sensitivity S at sample p, was calculated

(9) $$S\left(p\right)=\frac{\Delta R_{s}}{\Delta F}=\frac{R_{s}\left(p+1\right)-R_{s}\left(p\right)}{F\left(p+1\right)-F\left(p\right)}$$

and represented with the unit ($\;\frac{\Omega }{N}\;)$.

## 3. Results and Discussion

### 3.1. Morphological and Structural Analyses

The first part of the results characterise the textile substrates doped with different concentrations of GNPs. Such textiles will be integrated as the sensor active layer, and, for this purpose, it is important to identify the most adequate GNPs concentration. The optimum GNPs amount will allow the creation of a consistent path for the electrons flow, which, at the same time, does not compromise the intrinsic mechanical properties of a textile substrate. Note that 100% cotton woven fabric without any functionalisation will be, from now on, expressed by W.F, and a woven fabric functionalised only with the polymer matrix (0% GNP) will be used as the control sample (W.F.C). The same abbreviation logic is used for knitted fabric (K.F) and knitted fabric control (K.F.C).

**Figure 5:**
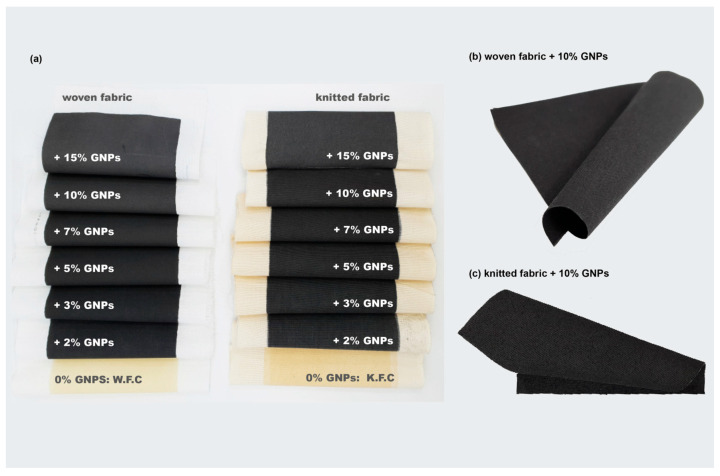
(**a**) Samples functionalised with different % of GNP and the control samples, in woven and knitted fabrics; (**b**) woven fabric functionalised with 10% GNP; (**c**) knitted fabric with 10% GNP.

Hereupon, the samples were analysed on different visual scales. Photographs allowed observing of a homogeneous coating, preserving the flexible structure of both textile substrates (see Figure 5).

**Figure 6:**
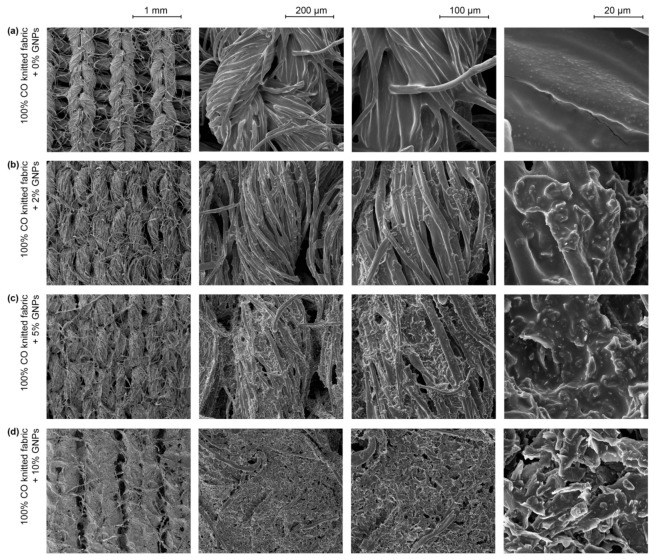
FESEM images of 100% CO knitted fabric: (**a**) 0% GNPs; (**b**) +2% GNPs; (**c**) +5% GNPs; (**d**) +10% GNPs.

**Figure 7:**
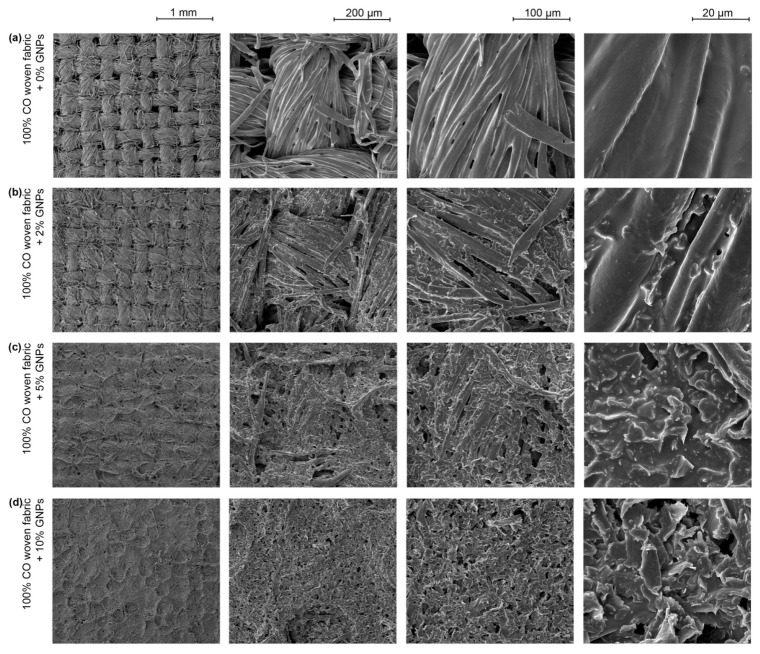
FESEM images of 100% CO woven fabric: (**a**) +0% GNPs; (**b**) +2% GNPs; (**c**) +5% GNPs; (**d**) +10% GNPs.

In another approach, FESEM images of the knitted (Figure 6) and woven fabrics (Figure 7) doped with 0%, 2%, 5% and 10% GNPs were obtained. Through Figure 6a and Figure 7a, it was possible to observe that the polymer matrix without GNPs homogeneously covered the fibres. Through Figure 6c and Figure 7c, in the less magnified images (with a scalebar of 1 mm and 200 μm), it was possible to see that from 5% GNPs, the coating started creating a consistent path over the fabrics (in both structures). With the increase in GNPs concentration to 10%, it was apparent that the coating comprehensively covered the warpwise and weftwise structure on woven fabric (Figure 6d). This behaviour can be justified by the topography of the woven fabric being more flattened to receive the deposition of a thin film when compared to a knitted structure, which is, commonly, made through an interlacing of loops. Meanwhile, more magnified images (with a 100 μm and 20 μm scale bar) revealed good dispersion between the polymeric matrix and the GNPs and good distribution of the GNPs between the fibres on both textile structures, with 5% and 10% nanoparticles. On the other hand, Figure 6b and Figure 7b show that the nanoparticle covering was sparse, which may compromise their effective electrical response.

Regarding changes in the textile structures, the deposition of an ink paste composed only by the polymeric matrix, that is, without any nanoparticle addition, increased the thickness of the samples by 12%. This value remained constant after the incorporation of up to 7% GNPs in knitted fabrics and 5% GNPs in woven fabrics, see Table 2. From 10% of GNPs up, this behaviour changed, and with the addition of 15% GNPs, there was an increase of 18% in the thickness of the knitted fabric and 20% in the thickness of the woven fabric, when compared to 100% cotton fabrics without any functionalisation process. Such values are higher than the typical 0.25 mm thickness found in the commercial polymers used in FSR sensors [12]. However, it should be noted that in this study, we are referring to textile substrates that, a priori, have a higher initial thickness value.

One factor is noteworthy in this structural result: the difference in thickness found in the functionalised woven fabrics, when compared to the same functionalisation conditions in the knitted structures, may indicate that the woven fabrics had a higher absorption capacity. Since the two textile structures were made of 100% cotton fibres, an intrinsically hydrophilic material, the structure of the textile itself, in this case, was the influencing factor for better absorption. Furthermore, this result is in line with previous studies that indicated a smooth textile surface as a crucial point for the good performance of their electro-conductive properties [24].

### 3.2. Physicochemical Characterisation

**Figure 8:**
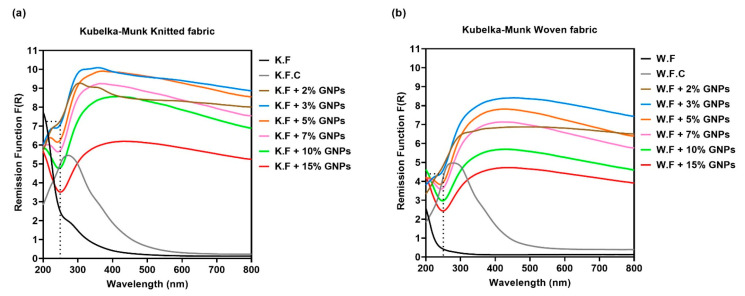
(**a**) GSDR spectra of non-functionalised and functionalised knitted fabrics with different GNPs %; (**b**) GSDR spectra of non-functionalised and functionalised woven fabrics with different GNPs %. The dotted line indicates the GNPs zone of absorption in the functionalised samples.

Figure 8a,b shows the Kubelka–Munk remission functions of cotton substrates without any functionalisation, doped with the polymer matrix (control sample), and coated with the inks in different GNPs %. All functionalised knitted and woven fabrics present an absorption zone in the UV-visible spectrum, characteristic of materials with a graphitic structure. The absorption zone at ~240 nm is attributed to the π → π * transition of the C-C aromatic bond, as indicated in literature [25]. It was also possible to observe that for samples coated with GNPs, there was a gradual decrease in the absorption band intensity proportional to the increase in the GNPs %. This decrease in the absorption intensity was related to an increase in the reflectance band. This phenomenon can be explained by the presence of GNPs in the textile samples, since they are nanoscale pigments with a high refractive index (*n*: 2.6–3) [26]. It is also possible to observe that for the same functionalisation conditions, the samples whose substrate was the woven fabric presented more intense reflectance bands. This result corroborates the previous morphological analysis, which indicated a superior presence of conductive material in this referred textile structure.

**Figure 9:**
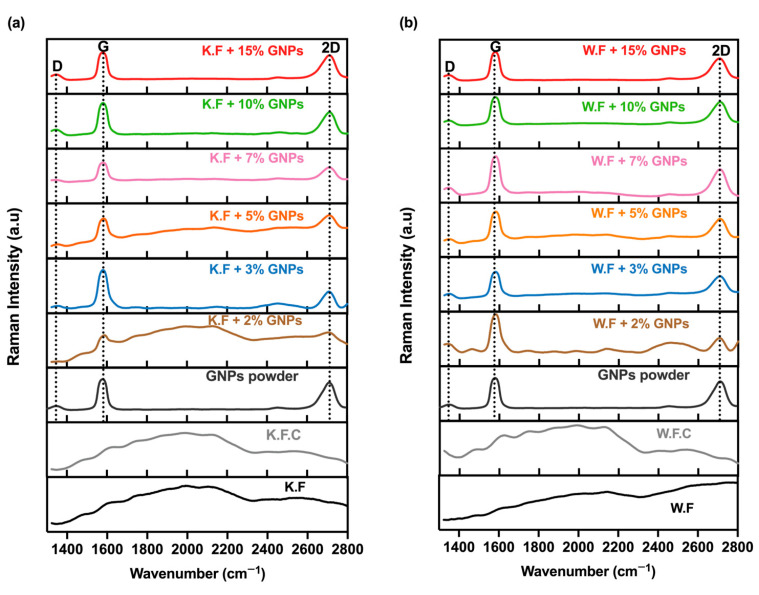
(**a**) Raman spectra of non-functionalised and functionalised knitted fabrics with different GNPs %; (**b**) Raman spectra of non-functionalised and functionalised woven fabrics with different GNPs %. The dotted line indicates the D, G and 2D peaks for the Graphene powder and all functionalised samples.

Raman spectroscopy is a qualitative and quantitative analysis method, which provides information about the structure and quality of the nanoparticles, as well as their degree of dispersion. This method is widely used and elected for the characterisation of carbon-based materials, taking into account that weak and inactive bands in the infrared, such as those referring to the stretching vibrations of the C=C bond, exhibit significant bands in the Raman spectrum [27]. That said, the three characteristic bands of carbon-based materials are the D, G and 2D:

The D band is related to the level of structural defects of nanoparticles, as it provides information about the chemical disorder that occurs due to the presence of sp^3^ hybridized carbon [28]. The G band is related to the presence of the sp^2^ hybridization plane vibrations of the carbon atoms [29]. On the other hand, the 2D band refers directly to graphite and provides information on its number of sheets [30]. As seen in Figure 9, the GNPs powder spectrum presented the D, G and 2D peaks, respectively, at approximately 1350 cm^−1^, 1580 cm^−1^ and 2700 cm^−1^. This result is in line with previous studies that used this type of nanoparticles [31].

Further information can be obtained through the direct relationship between the peaks’ intensity. In this sense, I_D_/I_G_ and I_2D_/I_G_ calculations were carried out for the GNPs powder spectrum. That said, since peak D was significantly lower than peak G, a value of 0.13 was identified for the I_D_/I_G_ ratio (see Table 3), which indicated few structural defects in the material used in these samples. In addition, 2D had a lower intensity than the G band, and the value for I_2D_/I_G_ obtained was 0.88, which suggests that the material used was mainly composed of multilayer graphene [21]. The same calculations were performed for all functionalised fabrics, as seen in Table 3. It was possible to observe that there was a gradual increase in the ID/IG ratio, concomitant with the increase in the GNPs % for samples in knitted structures. Such behaviour was not identified in woven fabric-based samples. However, from this result, what needs to be stated is that the functionalised samples showed a proportional correlation with the ID/IG and I2D/IG of the GNPs powder spectrum.

Figure 9a shows the spectra of the functionalised knitted fabrics, and Figure 9b refers to the spectra of doped woven fabrics. It is possible to observe the referred graphene powder peaks for all samples functionalised with different concentrations of GNPs. However, it should be noted that for samples doped with 2% GNPs, characteristic broad bands present in the control sample were evident and even more intense in the woven structure. This result is in agreement with the previous UV-Vis analyses which, while validating the deposition of graphene plates on both textile substrates, suggested that the knitted fabric absorbed a lower amount of ink paste when compared to the woven fabric, which was evident in both spectra for low GNPs concentrations.

### 3.3. Electrical Characterisation

The electrical resistance of the textile materials qualified them in terms of their applicability. A dry conventional textile structure made of natural fibres behaves as an electrical insulator [32]. After different functionalisation processes, these values vary, depending on the amount of conductive material deposited directly on the fibres or onto the textile structures. According to recent studies, for the construction of textile-based sensors, surface electrical resistance values in the order of 10^1^ Ω/sq were expected. From another perspective, for the development of an active material to be used specifically in pressure/force sensors following the piezoresistive principle, there was an empirical relationship between a greater sensitivity to mechanical stress with electrical resistance values in the order of kΩ. This reason is given by the factors that influence the electrical properties in polymer-based composites, namely, the polymer matrix, the type of nanoparticle, the processing methodology and the existence of post-treatments [17]. Hence, to obtain high electrical conductivity values, it was necessary to use a significant amount of conductive material. However, with the increased conductive material concentration deposited on a textile substrate, there was a consequent reduction in its deformation and flexibility capacity. Since these are crucial parameters directly related to the sensor’s sensitivity, whose electrical resistance is a function of mechanical deformation, it was necessary to identify a balance between the percentage of GNPs used in the samples to not compromise the intrinsic structural properties of the textiles.

**Figure 10:**
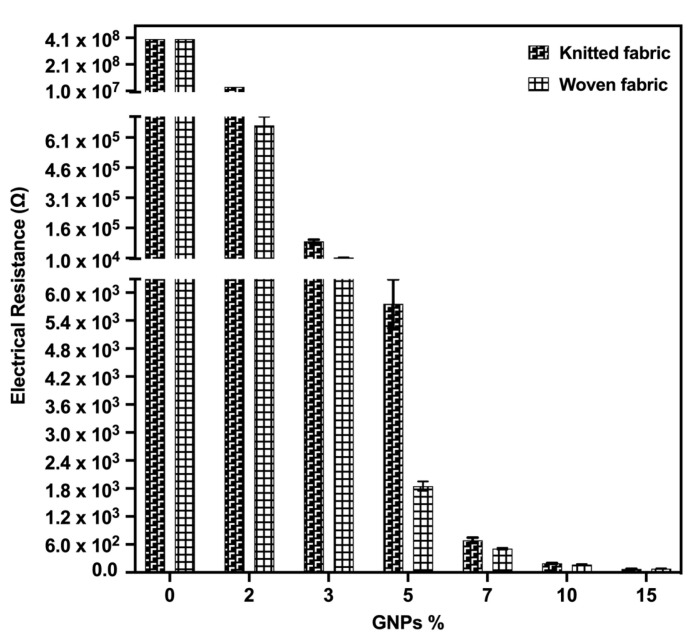
Electrical resistance values for samples functionalised with different GNPs%, in knitted and woven fabrics. The sample with 0% GNPs refers to the control sample, doped with the polymeric matrix only.

In view of the above, Figure 10 presents the electrical resistance values obtained in the functionalised samples with different GNPs %, when compared to the control samples only with the polymeric matrix. As expected, with an increasing concentration of conductive material, there was a progressive reduction in electrical resistance. This behaviour can be explained by the electrical path in the ink pastes in question, constituted by the two-dimensional contact (2-D) of the GNPs sheets in the polymeric matrix. It is noteworthy that the electrical resistance values obtained were lower than the ones obtained in similar studies using concentrations between 5% and 11% (*w*/*v*) of GNPs dispersed in PDMS [33]. This proves the good distribution of the GNPs in the used polymeric base, which had been optimised in this study. From 2% to 5% (*w*/*v*) GNPs, for both substrates, the samples reached values of semiconductor materials, in the order of kΩ. For 7% and 10% (*w*/*v*) GNPs, values in the order of hundreds of Ω were observed, and for 15% GNPs, the electrical resistance reached the order of Ω.

The woven fabrics samples showed lower electrical resistance values for all % GNPs, which indicated, once again, a higher presence of conductive material in woven when compared to the knitted fabrics.

Since they presented values of semiconductor materials, the samples with 2–5% of GNPs, in principle, stood as the ones with potentials to obtain the best performance concerning electrical resistance variation as a function of mechanical deformation. However, to validate this hypothesis and to have methods of comparison, textiles doped with 7% and 10% of GNPs were prepared for determining piezoresistive behaviour in addition to those samples. The sample with 15% GNPs was discarded at this stage, because the mechanical properties of the fabric in question were visibly compromised, even with a simple subjective evaluation.

#### Piezoresistive Behaviour

1. Knitted fabric sensors

Piezoresistive sensors, generally, are defined as “active” sensors, which depend on specific power circuits under the transducer technology. In addition, they are also used to measure dynamometric and geometric parameters, such as force, displacement and deformation, among others [13]. As previously detailed in this study, the sensors were connected to a signal conditioning circuit, which produced an output voltage (V) related to the sensor resistance (R_s_). It was notable that the knitted fabric sensors whose active material contained 2% and 3% GNPs, presented only noise when submitted to cyclic compression tests (data not shown). High noise levels in studies with carbon-based materials were justified by the abrupt change in connectivity of a sparser network (i.e., with a lower concentration of conductive materials), compared to the smoother changes in a dense network [34]. For the reason described above, these samples were discarded, as they did not show applicability.

**Figure 11:**
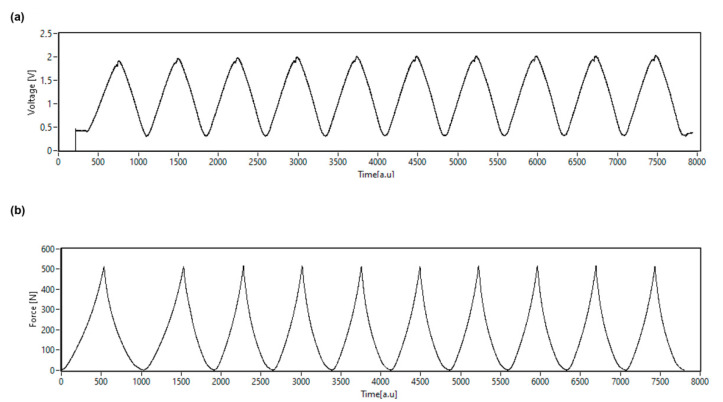
(**a**) the variation of voltage output with time for sample K.F + 10% GNPs; (**b**) the force variation over time for sample K.F +10% GNPs.

Figure 11 shows an example of the measurement carried out for sample K.F + 10% GNPs. It should be noted that during the piezoresistive test, two devices were used simultaneously to record the force and the voltage, with each device generating a different graph. In Figure 11a, the variation of voltage output with time is depicted, and this variation was produced by the force variation demonstrated in Figure 11b. In other words, what differentiates from Figure 11a to Figure 11b is that, for the same sample, (a) shows the variation of voltage output over time, while (b) represents the variation of force over time.

**Figure 12:**
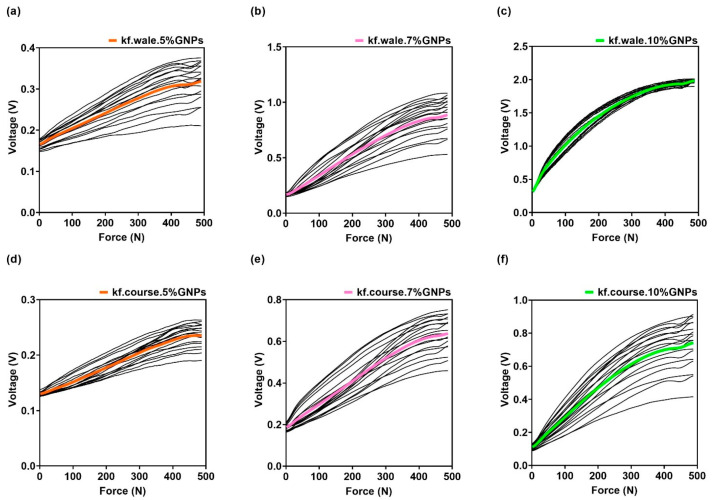
In black, Voltage signals acquired according to the sensor’s resistance, under 10 mechanical compression cycles for (**a**) knitted fabric sensor doped with 5% GNPs and assembled in the walewise direction; (**b**) knitted fabric sensor doped with 7% GNPs and assembled in the walewise direction; (**c**) knitted fabric sensor doped with 10% GNPs and assembled in the walewise direction; (**d**) knitted fabric sensor doped with 5% GNPs and assembled in the coursewise direction; (**e**) knitted fabric sensor doped with 7% GNPs and assembled in the coursewise direction; (**f**) knitted fabric sensor doped with 10% GNPs and assembled in the coursewise direction. The coloured curves represent the average values of voltage.

After synchronisation of force and voltage signals, the results shown in Figure 12 were obtained. An increase of output voltage was visible upon the increase in applied force, for knitted fabric sensors doped with 5%, 7% and 10% GNPs, in both the walewise and the coursewise directions. As previously shown by Equation (4), the voltage output (V_o_) is inversely proportional to the sensor resistance (R_s_); therefore, the resistance of the sensors was decreasing as the applied force increased.

The graphs in Figure 12 show the output of the signal conditioning circuit and allowed us to confirm the linearizing effect of the circuit, as well as to have a qualitative assessment of the voltage spread produced by the sensor over the 10 cycles. The sensitivity of the sensor could not be observed here, because the base resistance of the sensor was different from sample to sample, whilst the feedback resistor was the same; this placed the system in different operating points, with different gains, resulting in varying output voltage amplitudes. To compare the sensor’s sensitivity, the data regarding the values computed through Equation (9), shown later in this section, should be considered.

**Figure 13:**
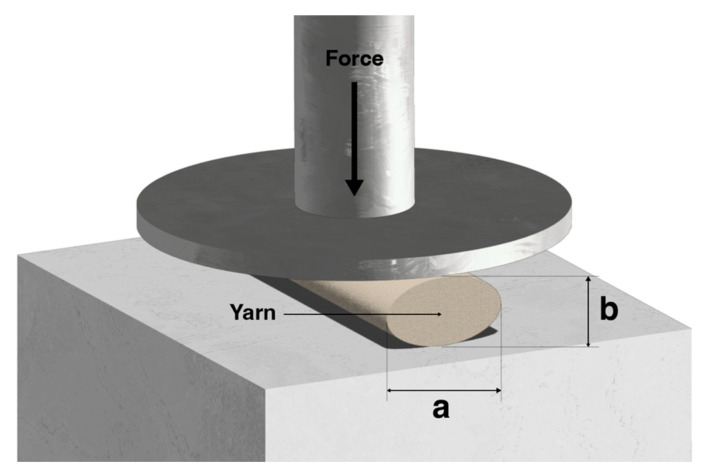
Cross-sectional view of a fibre being compressed, adapted from [32].

It should be noted that an important parameter to analyse the compressibility of a substrate is the compression resilience (RC), “a fundament which is the ratio of energy expended by the fabric in recovering from the deformation to the energy absorbed in deforming a fabric” [35]. That said, a previous study [36] showed that when a yarn is compressed, two distinct phenomena occurred in its diameter reductions. In the compression direction, it was called (b), and in the other direction, perpendicular to the compression, it was called (a), as observed in Figure 13. When the yarn was compressed, the (b) diameter was reduced, while it increased in (a). The authors named this yarn diameter reduction (b) as fibre “consolidation”, while the diameter increasing in (a) was called spreading. From the above, fibre “consolidation” behaviour entailed a bending tension, while fibre spreading was due to a friction phenomenon. However, the energy generated during the “consolidation” process improved the resilience compression (RC). On the other hand, the frictional phenomenon caused a loss of energy, and hence it reduced RC in the spreading process.

**Figure 14:**
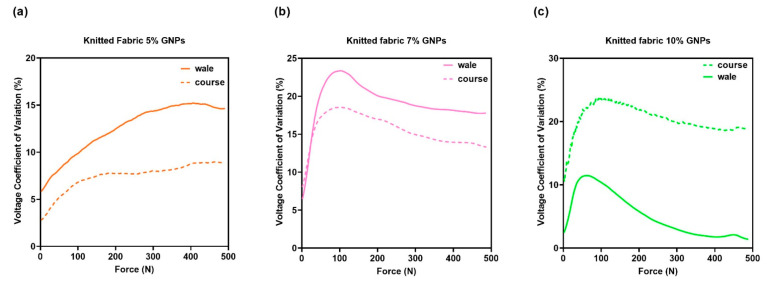
Voltage Coefficient of Variation (CV%) for (**a**) knitted fabric doped with 5% GNPs, whose active material was assembled towards the walewise (continuous line) and towards the coursewise directions (dotted line); (**b**) knitted fabric doped with 7% GNPs, whose active material was assembled towards the walewise (continuous line) and towards the coursewise directions (dotted line); (**c**) knitted fabric doped with 10% GNPs, whose active material was assembled towards the walewise (continuous line) and towards the coursewise directions (dotted line).

In this study, it was possible to make a correlation between the voltage dispersion in terms of average value (CV%) and the knitted fabric’s ability to return to its initial state after compression. Figure 14 shows the output voltage coefficient of variation over the force range tested. In general, it can be understood that a lower voltage CV% for fabric cmeans that the fabric has a higher compression resilience value where it retains its shape. Hence, it can be observed from Figure 14a,b in the graphs that the CV% for knitted fabric in the coursewise direction was lower than that in the walewise direction. Thus, it can be concluded that the fabric had a larger RC in the coursewise in comparison with the walewise. In line with this relationship, a recent study proved that fibre elongation has a direct influence on the RC factor [37], while others reported that elastic recovery for a knit structure occurs more strongly in the coursewise direction in comparison to the walewise direction [38]. However, the yarn direction is not relevant anymore at amounts of GNPs above 10%. This happens because a film is formed, as can be seen in the microscopy images in Figure 6a, suggesting that the piezoresistive behaviour relies on the percolation theory and no longer on the intrinsic mechanical deformation of the textile material.

**Figure 15:**
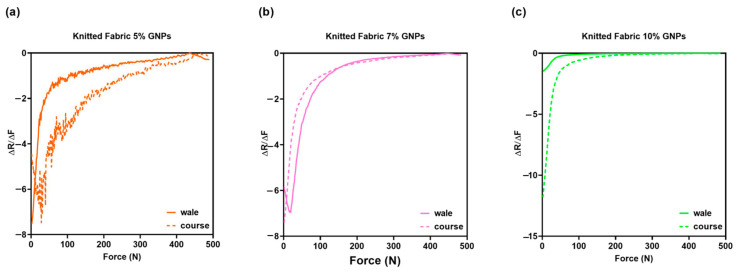
Sensitivity (S) of (**a**) knitted fabric doped with 5% GNPs, whose active material was assembled towards the walewise (continuous line) and towards the coursewise directions (dotted line); (**b**) knitted fabric doped with 7% GNPs, whose active material was assembled towards the walewise (continuous line) and towards the coursewise directions (dotted line); (**c**) knitted fabric doped with 10% GNPs, whose active material was assembled towards the walewise (continuous line) and towards the coursewise directions (dotted line).

For a nonlinear transfer function, the sensitivity was not a fixed number as for the linear relationship [35]. As previously explained, the aforementioned pressure sensors presented different behaviour in the walewise and coursewise directions, a phenomenon that is reflected in distinct sensitivity responses, as shown in Figure 15. It should be noted that the knitted sensors doped with 5% and 10% GNPs, positioned in the coursewise direction, presented a larger sensitivity, when compared to the sensors positioned in the walewise direction. For higher forces (200–500 N), there was no significant difference in the sensitivity behaviour related to yarn direction in knitted sensors doped with 7% and 10% GNPs.

2. Woven fabric sensors

In both fabric substrates, the 2% GNPs solution was inadequate for a valid electrical response. However, although still with significant noise, the woven fabric sensor doped with 3% GNPs was amenable to characterisation by showing a relatively constant electrical response to mechanical deformation, which did not happen for the knitted fabrics presented before.

**Figure 16:**
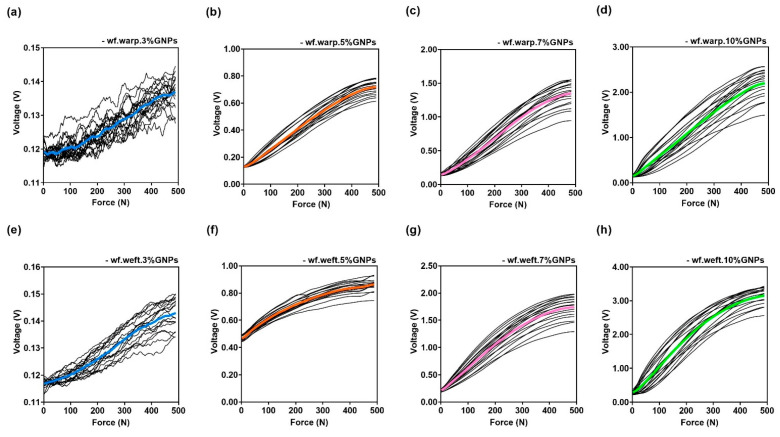
In black, Voltage signal acquired according to the sensor’s resistance, under 10 mechanical compression cycles for (**a**) woven fabric sensor doped with 3% GNPs and assembled in the warpwise direction; (**b**) woven fabric sensor doped with 5% GNPs and assembled in the warpwise direction, (**c**) woven fabric sensor doped with 7% GNPs and assembled in the warpwise direction; (**d**) woven fabric sensor doped with 10% GNPs and assembled in the warpwise direction; (**e**) woven fabric sensor doped with 3% GNPs and assembled the weftwise direction; (**f**) woven fabric sensor doped with 5% GNPs and assembled in the weftwise direction; (**g**) woven fabric sensor doped with 7% GNPs and assembled in the weftwise direction; (**h**) woven fabric sensor doped with 5% GNPs and assembled in the weftwise direction. The coloured curves represent the average values of voltage: 3% GNPs are represented by the colour blue, 5% GNPs are represented by the colour orange, 7% GNPs are represented by the colour pink, 10% GNPs are represented by the colour green.

These results validate the physical and chemical characterisations, where a larger GNPs absorption was visible for the woven substrate when compared to the knitted fabric. Figure 16 presents the piezoresistive behaviour of the woven fabric sensors doped with 3%, 5%, 7% and 10% GNPs, and, as previously identified through the knitted samples, it can be seen that there was an increase in output voltage, concomitant with an increase in applied force.

**Figure 17:**
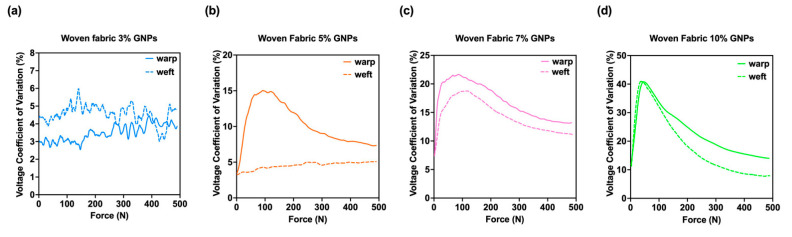
Voltage Coefficient of Variation (CV%) for (**a**) woven fabric doped with 3% GNPs, whose active material was assembled towards the walewise (continuous line) and towards the coursewise directions (dotted line); (**b**) woven fabric doped with 5% GNPs, whose active material was assembled towards the walewise (continuous line) and towards the coursewise directions (dotted line); (**c**) woven fabric doped with 7% GNPs, whose active material was assembled towards the walewise (continuous line) and towards the coursewise directions (dotted line); (**d**) woven fabric doped with 10% GNPs, whose active material was assembled towards the walewise (continuous line) and towards the coursewise directions (dotted line).

Figure 17 shows that the samples whose active layer was assembled in the weftwise direction had the lowest spread, expressed by the coefficient of variation. These results are in agreement with the previous results of knitted sensors, as a lower CV% was achieved in both situations for the yarns in the vertical direction. The only exception was in the sample with 3% GNPs, which reflects a high electrical noise in the signals. It should be noted that the greatest relative deviation intensity was in the range between 0–200 (N). However, in this referred force range, the sample with 10% GNPs presented no significant difference regarding the direction of the sample.

**Figure 18:**
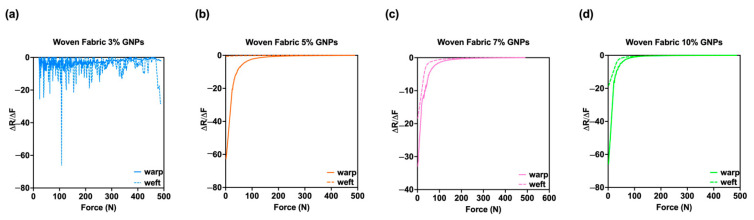
Sensitivity (S) of (**a**) woven fabric doped with 3% GNPs, whose active material was assembled towards the walewise (continuous line) and towards the coursewise directions (dotted line) both are represented by the colour blue; (**b**) woven fabric doped with 5% GNPs, whose active material was assembled towards the walewise (continuous line) and towards the coursewise directions (dotted line), both are represented by the colour orange; (**c**) woven fabric doped with 7% GNPs, whose active material was assembled towards the walewise (continuous line) and towards the coursewise directions (dotted line), both are represented by the colour pink; (**d**) woven fabric doped with 10% GNPs, whose active material was assembled towards the walewise (continuous line) and towards the coursewise directions (dotted line), both are represented by the colour green.

From another perspective, Figure 18 shows a higher sensitivity for all woven sensors whose active material was positioned in the warpwise direction. Regarding this result, it is noteworthy that in the current woven fabric composite, the warpwise and weftwise yarns were of different cross-section shapes and crimp ratios. The applied tension displacements in warpwise and weftwise directions were calculated based on the corresponding crimp ratios, and the displacement of compression was constrained to match the current thickness [36]. In turn, the term crimp ratios refers to the level of undulation in one direction of the woven fabric, and, in this sense, it is evident that yarns in the warpwise direction show less undulation than those in the weftwise direction. Thus, a better path for the passage of the electrons is created. On the other hand, the yarn compressibility is related to their straightening, followed by the change in their diameter. As explained earlier, the friction caused by this thickness reduction made the fibres more compacted and increased the number of contact points between them. Therefore, due to the superior crossing zone per unit area created through the warpwise direction, a larger resistance signal variation range was identified in this referred direction, resulting in a larger sensitivity.

In line with the above, the warpwise density (32 cm^−1^) was superior to the weftwise density (29 cm^−1^) in the control fabric. Furthermore, we should also assume surface interruptions of series-connected elements, with a simultaneous significant increase in the number of parallel joints in the yarn structure, so it is logical that the total resistance at the measured yarn length decreases [39].

As evidenced earlier, sensitivity is an important parameter to distinguish pressure sensors. However, it should be noted that this parameter can be adjusted by amplification, as indicated in the literature [40,41], while the coefficient of variation cannot. Therefore, evaluating the textile mechanical behaviour and identifying the factors that lead to presenting lower CV% could indicate the parameter that will contribute to a stabler textile-pressure sensor response.

## 4. Conclusions

The main goal of this study was to investigate the influence of the fabric orientation on the performance of knitted and woven pressure sensors.

FESEM, Raman spectroscopy and GSDR analyses not only validated the presence of GNPs on the textile substrates, but they also showed that the flattened topography of the woven fabric and its lower thickness presented a higher absorption capacity, with lower electrical resistance values.

From a qualitative perspective, the piezoresistive tests showed that the geometric deformation of the substrates and the direction of the yarns generated different electrical responses. To sum up:

- In the transversal direction, coursewise for knitted and weftwise for woven fabrics, the friction phenomenon caused a lower CV%, suggesting that there is a correlation between higher RC and lower CV%;
- Crimp ratios and superior crossing zone per unit area provided by its warpwise density contributed to a higher sensitivity in woven fabrics, in the warpwise direction.

As previously evidenced, sensitivity is a parameter that can be adjusted by amplification, but the coefficient of variation cannot. Therefore, given the results obtained, woven fabrics showed lower CV% than knitted fabrics. In addition, woven fabric doped with 5% GNPs presented the lowest CV% in the weftwise direction, so it is the most indicated for a more accurate measurement of mechanical action.

Therefore, to get a better design process for textile piezoresistive sensors, it is necessary to have an understanding of the anisotropic behaviour of textile substrates under compression, in addition to what the literature commonly indicates. This behaviour can be intrinsic to the substrate itself, or it can be changed according to the amount of polymeric material used. Each sample must be analysed individually, in order to be able to identify an active textile material with the lowest CV%.

Furthermore, we believe that if the sensor is intrinsically placed in the fibres, the structural parameters of the textile substrates can be orchestrated more precisely, improving the sensor’s performance.

Regarding applicability, such sensors can be used close to the skin as a qualitative input of body pressure, both to identify disturbances and enhance human–machine interaction, but also to measure large forces, specifically those between 200 and 500 N.

For future works, we expect to develop a quantitative study on geometric parameters that influence the electrical response of knitted and woven fabrics under compression conditions.

## Figures and Tables

**Table 1:** Structural characteristics of woven and knitted and woven fabric.

Knitted Fabric		Woven Fabric	
Walewise density	12 cm^−1^	Warpwise density	32 cm^−1^
Coursewise density	26 cm^−1^	Weftwise density	29 cm^−1^
Wale yarn	20.25 Tex	Warp yarn	19.59 Tex
Course yarn	20.25 Tex	Weft yarn	17.31 Tex
Loop length	0.22 cm	-	-
Thickness	0.48 mm	Thickness	0.21 mm
Mass per unit surface	145.6 g/m^2^	Mass per unit surface	122 g/m^2^

**Table 2:** Textile samples and their thickness values.

Sample	Thickness (mm) ± S.D	Sample	Thickness (mm) ± S.D
K.F	0.50 ± 0.006	W.F	0.25 ± 0.011
K.F.C	0.57 ± 0.00	W.F.C	0.28 ± 0.009
K.F.C + 2% GNPs	0.56 ± 0.007	W.F.C + 2% GNPs	0.28 ± 0.005
K.F.C + 3% GNPs	0.57 ± 0.008	W.F.C + 3% GNPs	0.28 ± 0.005
K.F.C + 5% GNPs	0.57 ± 0.011	W.F.C + 5% GNPs	0.28 ± 0.004
K.F.C + 7% GNPs	0.57 ± 0.007	W.F.C + 7% GNPs	0.29 ± 0.004
K.F.C + 10% GNPs	0.58 ± 0.010	W.F.C + 10% GNPs	0.30 ± 0.006
K.F.C + 15% GNPs	0.60 ± 0.010	W.F.C + 15% GNPs	0.35 ± 0.028

**Table 3:** I_D_/I_G_ and I_2D_/I_G_ values for GNPs Powder and functionalised knitted and woven fabric with different GNPs %.

Sample	I_D_/I_G_	I_2D_/I_G_	Sample	I_D_/I_G_	I_2D_/I_G_
GNPs Powder	0.13	0.88	-	-	-
K.F + 2% GNPs	0.04	1.10	W.F + 2% GNPs	0.22	0.44
K.F + 3% GNPs	0.06	0.44	W.F + 3% GNPs	0.22	0.59
K.F + 5% GNPs	0.13	1.11	W.F + 5% GNPs	0.15	0.79
K.F + 7% GNPs	0.17	0.75	W.F + 7% GNPs	0.19	0.67
K.F + 10% GNPs	0.23	0.73	W.F + 10% GNPs	0.21	0.84
K.F + 15% GNPs	0.28	1.01	W.F + 15% GNPs	0.22	0.80
